# Synthesis and Characterization of Encapsulated Nanosilica Particles with an Acrylic Copolymer by *in Situ* Emulsion Polymerization Using Thermoresponsive Nonionic Surfactant

**DOI:** 10.3390/ma6093727

**Published:** 2013-08-28

**Authors:** Mostafa Yazdimamaghani, Tannaz Pourvala, Elaheh Motamedi, Babak Fathi, Daryoosh Vashaee, Lobat Tayebi

**Affiliations:** 1 School of Material Science and Engineering, Helmerich Advanced Technology Research Center, Oklahoma State University, Tulsa, OK 74106, USA; E-Mail: mostafa.yazdimamaghani@okstate.edu; 2 Organic Chemistry and Polymer Research Laboratory, Iranian Institute of Research and Development in Chemical Industries (ACECR), Tehran, Iran; E-Mails: tannazafghani@yahoo.com (T.P.); babak.poly@gmail.com (B.F.); 3 Department of Chemistry, Tarbiat Modares University, P.O. Box 14155-175, Tehran, Iran; E-Mail: motamedi@modares.ac.ir; 4 School of Electrical and Computer Engineering, Helmerich Advanced Technology Research Center, Oklahoma State University, Tulsa, OK 74106, USA; E-Mail: daryoosh.vashaee@okstate.edu; 5 School of Chemical Engineering, Oklahoma State University, Stillwater, OK 74078, USA

**Keywords:** silica nanoparticles, thermoresponsive nonionic surfactant, cloud point, emulsion polymerization

## Abstract

Nanocomposites of encapsulated silica nanoparticles were prepared by *in situ* emulsion polymerization of acrylate monomers. The synthesized material showed good uniformity and dispersion of the inorganic components in the base polymer, which enhances the properties of the nanocomposite material. A nonionic surfactant with lower critical solution temperature (LCST) was used to encapsulate the silica nanoparticles in the acrylic copolymer matrix. This *in situ* method combined the surface modification and the encapsulation in a single pot, which greatly simplified the process compared with other conventional methods requiring separate processing steps. The morphology of the encapsulated nanosilica particles was investigated by dynamic light scattering (DLS) and transmission electron microscopy (TEM), which confirmed the uniform distribution of the nanoparticles without any agglomerations. A neat copolymer was also prepared as a control sample. Both the neat copolymer and the prepared nanocomposite were characterized by Fourier transform infrared spectroscopy (FTIR), thermal gravimetric analyses (TGA), dynamic mechanical thermal analysis (DMTA) and the flame resistance test. Due to the uniform dispersion of the non-agglomerated nanoparticles in the matrix of the polymer, TGA and flame resistance test results showed remarkably improved thermal stability. Furthermore, DMTA results demonstrated an enhanced storage modulus of the nanocomposite samples compared with that of the neat copolymer, indicating its superior mechanical properties.

## 1. Introduction

The field of inorganic-organic nanocomposites is growing rapidly, because such hybrid materials can possess combined properties of both the incorporated inorganic materials and the base polymers. Among all the hybrid materials, nanocapsules are especially interesting, due to their capability in making a diverse range of new materials for different applications [1]. A wide variety of colloidal inorganic materials has been used in polymer nanocomposites, including silica [2], titanium dioxide [3], copper oxide [4], magnetic oxide [5], aluminum hydroxide [6], silver [7], clay [8] and carbon black [9]. Among them, silica is the most studied material, because hybrid structures of silica and polymer have excellent physical reinforcement, high thermal resistance, high flexibility, high gas permeability and low surface energy, due to the incorporation of silica [10,11]. Uniform dispersion of nanosilica in polymers can improve the strength, the abrasion-resistance, the aging-resistance and the climate-resistance of the polymer [12,13], as well. Therefore, they have been used in thermal insulators, bioactive supports, paints, plastics, rubbers, coatings, drug delivery systems and composite materials [14,15,16,17]. Among different structures of silica nanoparticles, silica nanoparticles with a highly ordered mesoporous structure (MSNs), which can be synthesized using triblock copolymers with high poly(alkylene oxide) segments in acid media, can be used in drug delivery systems by loading of different guest molecules, such as peptides, proteins, anticancer agents and genetic material [18,19].

Since the properties of the resulting composite materials are expected to be influenced by the original particle morphology, much effort has been applied to produce nanocomposite particles with controlled shapes. Hence, the silica-polymer nanocomposite particles with various morphologies, such as silica (core)-organic material (shell) [20], organic (core)-silica (shell) [21], raspberry-like [22], snowman-like [23], daisy-shaped, multipod-like [24] and raisin bun-like [25], have been produced by different techniques. These nanocomposite particles are commonly produced by incorporation of colloidal silica in heterogeneous polymerizations, such as emulsion, dispersion and suspension [26,27,28]. Although these methods have been reported to produce fine nanocomposites, in order to prepare organic-inorganic nano-hybrids, emulsion polymerization is the preferred route to produce nanocomposites, especially based on acrylic or styrenic polymers. This is mainly due to its ease of processability and the possibility to distribute small concentrations of nanoparticles into nanoscale-independent matrices [29,30].

The properties of nanocomposites are greatly influenced by the dispersion degree of the inorganic components in the base polymer. The agglomeration and poor dispersion of the filler in the polymer matrix can reduce the anticipated enhancement of properties, like the strength, adhesion and durability and abrasion resistance of the material. The agglomeration may even worsen the properties of the nanocomposite in comparison with the pristine polymer [31]. The dispersion of silica nanoparticles in the polymer matrix is confronted by the lipophobic properties of the silica nanoparticles. The silica nanoparticles have negative surface charge, due to the existence of the silanol-type hydroxyl groups on their surface. The surface charge of the silica nanoparticles makes them highly hydrophilic, which causes their agglomeration during mixing [32]. In addition, the incompatibility of the hydrophilic nanoparticles and the hydrophobic matrix will result in poor interfacial interaction that can weaken the mechanical properties [33]. In order to achieve a good dispersion of the silica nanoparticles and to increase the interface adhesion with the polymer, physical and chemical approaches have been used to modify the silica surface [34].

In chemical modification, silica nanoparticles are often treated with silane coupling agents to enhance their dispersibility [35,36]. Grafting of polymer chains to a nanosilica surface is also an effective chemical process to increase the hydrophobicity of the particles [37,38,39].

Surface modification based on physical interaction is usually applied by adsorbance of surfactants or macromolecules onto the surface of the silica particles. It can be easily incorporated into a polymer matrix [31,40,41,42,43,44]. A surfactant can absorb to the surface of silica by electrostatic interaction of its polar group, which can reduce the interaction among the silica particles, preventing the agglomeration.

Miyamoto and co-workers [29] reported a simple method to modify silica nanoparticles using poly(ethylene oxide) type reactive nonionic surfactants with an allyl group and an oligo(oxyethylene) chain (NE-10) above their cloud point (40 °C). They used this method in the polymerization to induce the deposition of the surfactant onto the silica sol interface, which enhances the affinity of the silica surface to the organic materials. Recently, with a similar approach, Dashtizadeh *et al*. [45] prepared nanocomposite emulsion acrylic resins with nanosilica particles and studied the effect of changing copolymers and silica contents of latexes on the improvement of the pendulum hardness, solvent resistance and glossiness.

Over the last two decades, stimuli-responsive macromolecules (*i.e.*, pH-, thermo-, photo-, chemo-, and bio-responsive polymers) have attracted the scrutiny and considerable interest of materials and nanotechnology scientists. In particular, thermoresponsive macromolecules that have cloud point or lower critical solution temperature (LCST) are useful for a wide variety of applications. Thermoresponsive macromolecules switch from hydrophilic to hydrophobic states above LCST in response to temperature changes, which makes them dehydrate and aggregate. Herein, we have synthesized the encapsulated nanosilica through simple *in situ* emulsion polymerization of acrylic monomers using poly(ethylene oxide)-type without an allyl group nonionic surfactant, such as nonyl phenol polyethylene glycol ether (NP-7), above its cloud point (25 °C) for modification of nanosilica surface. The produced nanocomposites have been characterized by FTIR, DLS, TEM, TGA, DMTA and the flammability test. The analysis showed the encapsulation of silica nanoparticles. Furthermore, different properties of the nanocomposites were compared with the neat acrylic resin without nanosilica.

## 2. Results and Discussion

### 2.1. Encapsulation of Silica Nanoparticles with Acrylic Copolymer

**Figure 1:**
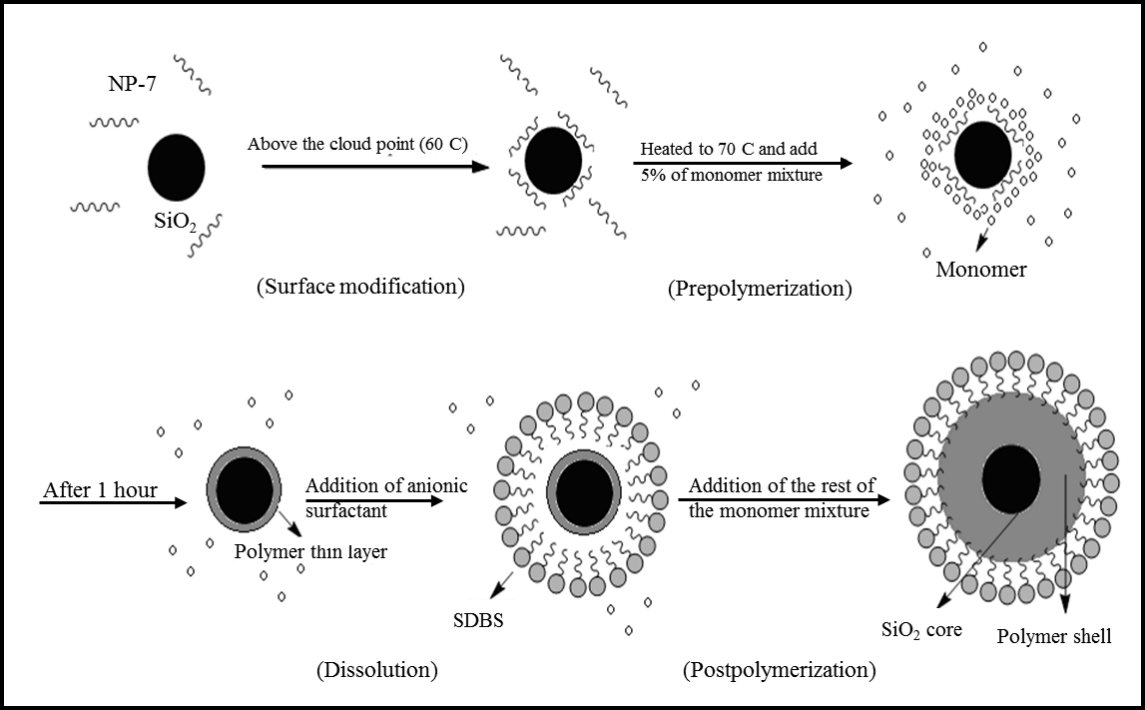
Schematic mechanism of the formation process of encapsulated nanosilica particles with the acrylic copolymer by *in situ* emulsion polymerization using thermoresponsive nonionic surfactant.

A nanosilica particle has negative surface charges, due to SiO- and silanol-type hydroxyl groups. We used nonionic surfactant (nonyl phenol polyethylene glycol ether) above its LCST in the emulsion polymerization of acrylate monomer in the presence of the silica nanoparticles. This technique can modify the hydrophilic surface of the silica sol and enhance its affinity to the organic materials. The emulsion polymerization consists of four steps: surface modification, prepolymerization, dissolution and postpolymerization [29]. Figure 1 schematically shows the formation procedure of encapsulated nanosilica particles with the acrylic copolymer.

At the first step, the colloidal silica was mixed with a nonionic surfactant (NP-7, cloud point <25 °C) at 60 °C. At this temperature, which is higher than the cloud point temperature, the surfactant affinity to water is almost lost. Therefore, a large part of the surfactant molecules is adsorbed onto the silica particles as the poly(oxyethylene) block in the surfactant can bond with the hydroxysilyl groups through hydrogen bonding. As a result of switching surfactant affinity from the hydrophilic to the hydrophobic state above the LCST in response to the temperature change, the turbidity of the mixture slightly increased. The surfactant layer formed on the surface of the silica nanoparticles gives them a hydrophobic property, which makes the polymerization on the surface of silica particles possible.

At the second step, the system was heated to 70 °C, and a small amount of the monomer mixture was added into the suspension; the system was kept for 1 h at this temperature with vigorous stirring without a significant change in the appearance of the suspension. Due to the strong hydrophobicity of the monomers, we hypothesize that part of the monomers entered into the nonionic surfactant layer of the modified silica, forming a fixed organic shell around the silica particles.

At the third step, after adding the anionic surfactant, sodium dodecylbenzenesulfonate (SDBS), the turbidity of the system was partially paled. The anionic surfactant molecules coated the nanocomposites, giving them the affinity to water. Therefore, the dispersion of the nanocomposite particles in water was enhanced, which explained the change in the color of the solution.

At the final step, the rest of the monomer mixture was added into the system. The monomer liquid entered into the anionic surfactant layer and covered the silica particles. An initiating radical penetrated into the particle and started the polymerization, like in the usual case of emulsion polymerization, to produce the desired nanocomposite system.

### 2.2. FTIR Analysis

**Figure 2:**
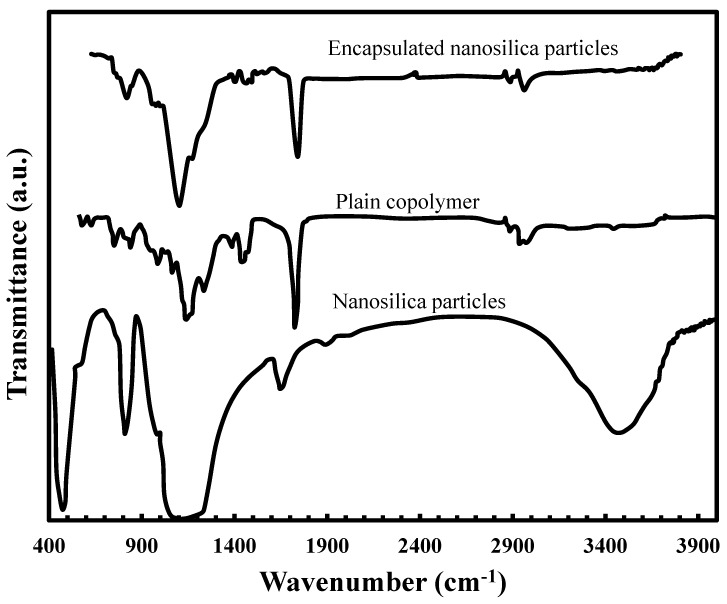
Fourier transform infrared spectroscopy (FTIR) spectra of nanosilica particles, plain copolymer and encapsulated nanosilica particles with the acrylic copolymer.

Since the present work aims to investigate the effect of the encapsulation of the nanosilica particles with an acrylic copolymer, the variation in the chemical structure of the particles should be known at the very beginning of the analysis. The FTIR spectra of the silica nanoparticles, plain copolymer and the synthesized nanocomposites are shown in Figure 2. The FTIR spectra of the silica nanoparticles and the synthesized nanocomposite clearly showed the effective modification of the silica surface with acrylic copolymer. The spectra of Figure 2 exhibited a number of characteristic spectral bands, such as: the peaks at 1,140,808 and 473 cm^−1^ due to the asymmetric stretching vibration, symmetric stretching vibration and bending vibration of Si–O–Si, respectively, which are the specific bands of the silica nanoparticles [46,47,48]. The peak at 960 cm^−1^ is ascribed to the stretching vibration of Si–OH [46,49]. The broad band around 3480 cm^−1^ can be attributed to absorbed water and to H-bonded silanol (OH) groups [46,47,48]. In the encapsulated silica nanoparticles, the tether between the silica and the polyacrylate chains caused the band absorptions corresponding to the C–OH and C–O–C groups of the polyacrylate chains, which are significantly overlapped with the Si–O–Si group of the silica, as shown in the spectral range of 1000–1400 cm^−1^. However, the peaks that appeared at 1740 and 1440 cm^−1^, respectively, referred to the stretching vibration of carbonyl group and the bending vibration peak of C-H in acrylic copolymer. In addition –CH_3_ and –CH_2_ absorbance peaks for the copolymer around 2900 to 3100 cm^−1^ are observed for both neat copolymer and prepared nanocomposite [43,50,51]. The FTIR spectrum showed a decrease in the adsorption band intensity at 3480 cm^−1^, indicating that approximately all of the silica nanoparticles had been encapsulated by the polymer. The absorption spectrum of silica nanoparticles, plain copolymer and the nanocomposite proved that silica nanoparticles were successfully coated with the copolymer.

### 2.3. Particle Size Distribution

**Figure 3:**
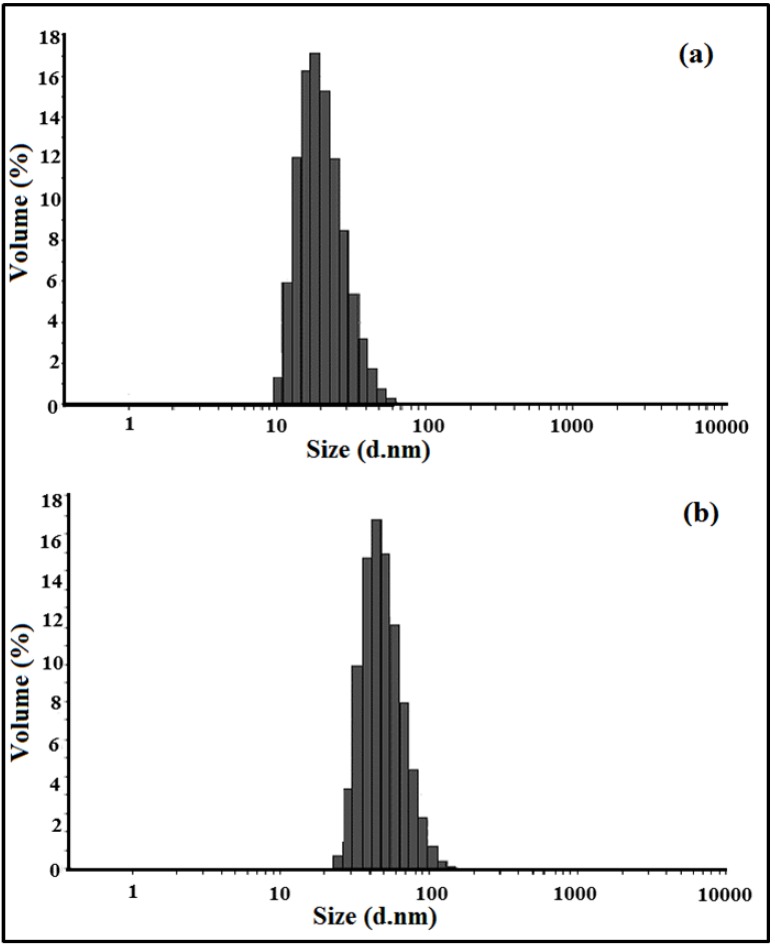
Dynamic light scattering (DLS) results obtained for (**a**) nanosilica sol; and (**b**) prepared nanocomposite.

The particle size distributions of the nanocomposite and the nanosilica sol are presented in Figure 3. The average diameter of the silica sol and the nanocomposite were estimated as 27 nm and 59 nm, respectively. The size of the nanocomposite particles were almost twice the size of the silica particle, but the majority of nanocomposite particles remained under 100 nm. The increase of size confirms that the copolymer of MMA/BA coated the surface of the nanosilica particles [51]. The polydispersity (*i.e.*, the square of the ratio of the standard deviation to the mean diameter size) of the nanocomposite was less than 0.1, which agrees with the notion that the monomers of MMA/BA and the surfactants are adsorbed on the surface of the nanosilica, and the surfactant acted as micelles imposing the polymerization taking place around the nanosilica. In fact, the second step in the emulsion polymerization (prepolymerization) leads to the uniform particle size and the narrow polydispersity.

### 2.4. Morphological Studies

**Figure 4:**
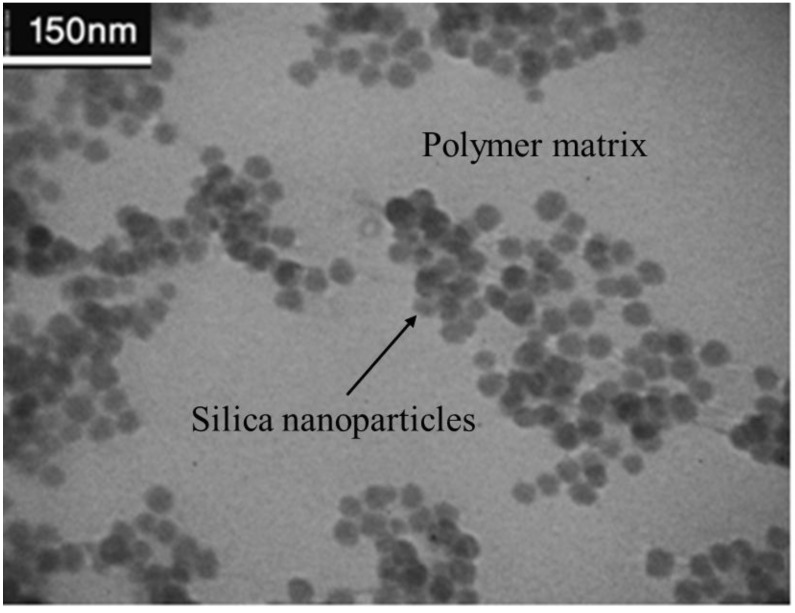
Transmission electron microscopy (TEM) micrographs of the composite latex particles containing silica/acrylate polymer core-shell morphology.

The morphology of synthesized nanocomposite was studied using the TEM as shown in Figure 4. The electronic density of silica is much larger than that of polymeric matrix; therefore, the nanosilica appeared black or dark grey. The pale parts were acrylate polymer as a continuous phase enwrapping the silica particles. It was observed that silica particles are wrapped by the acrylate polymer and distributed uniformly in the polymer matrix. The TEM micrograph verifies the absence of aggregation, due to using the *in situ* emulsion polymerization of acrylate monomers with a nonionic surfactant with appropriate LCST property. It is noteworthy that in this procedure, there is no separate process step for the surface modification of the silica particles.

### 2.5. Thermal Analysis

**Figure 5:**
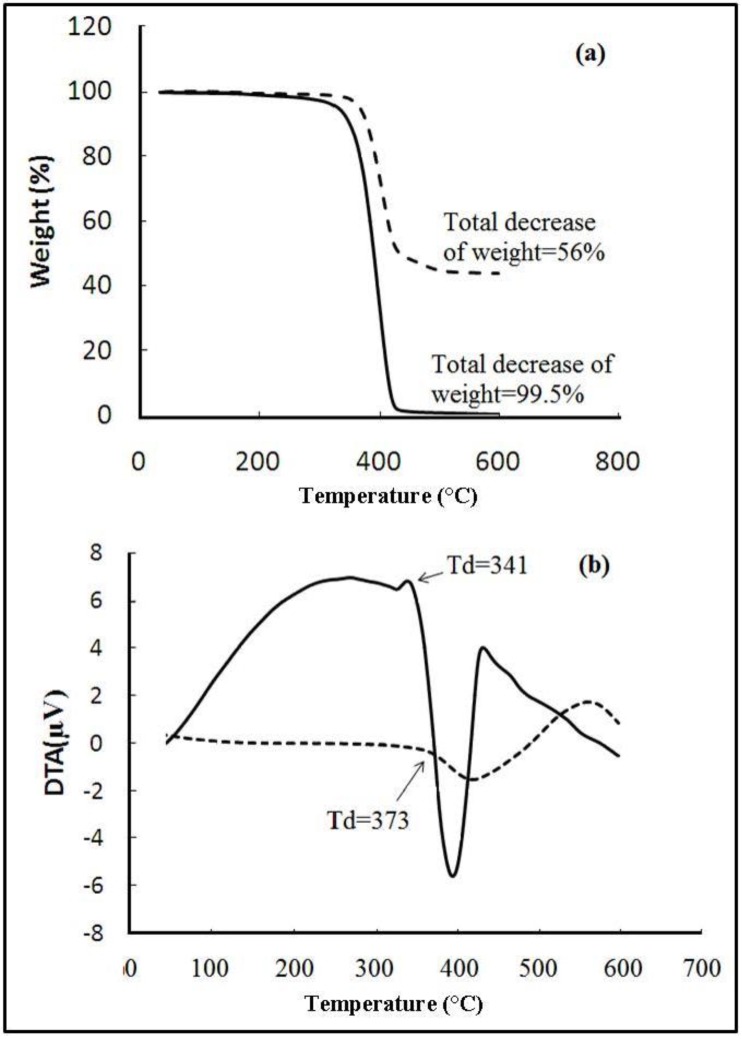
**(a)** Thermal gravimetric analyses (TGA) curves; and **(b)** DTA thermographs nanocomposite (dashed line) and plain copolymer (solid line).

Figure 5a shows the TG/DTA results of the neat and the acrylate-silica nanocomposite synthesized by the emulsion polymerization. Figure 5a shows the residual weight of the nanocomposite and the neat copolymer after the decomposition at high temperature (*T* > 500 °C). Subtracting the residual weight of the nanocomposite (dashed line) from that of the copolymer (solid line) gives the silica content. As can be seen in Figure 5a, silica can significantly increase the residual weight of the acrylic latex at high temperatures from 0.5 to 44 percent, which is quite consistent with the content of the silica in the nanocomposite. As shown in Figure 5b, it seems that the presence of the nanosilica can cause a significant increment in the decomposition temperature (Td). DTA curves show that the decomposition temperature (Td) for the nanocomposites is higher (373 °C) than that of the pure acrylic latex (341 °C). Comparing the TG and DTA curves for both systems, one can determine that the degradation process (exothermic peaks), which goes along with the weight reduction, started just after the thermal decomposition. It is worth mentioning that the broad DTA peaks in the temperature region lower than 300 °C correspond to the melting and decomposition of nonionic emulsifier (nonyl phenol polyethylene glycol ether) used in the preparation of nanocomposites, as well as the water absorbed at the surface of samples during analysis.

### 2.6. DMTA Analysis

**Figure 6:**
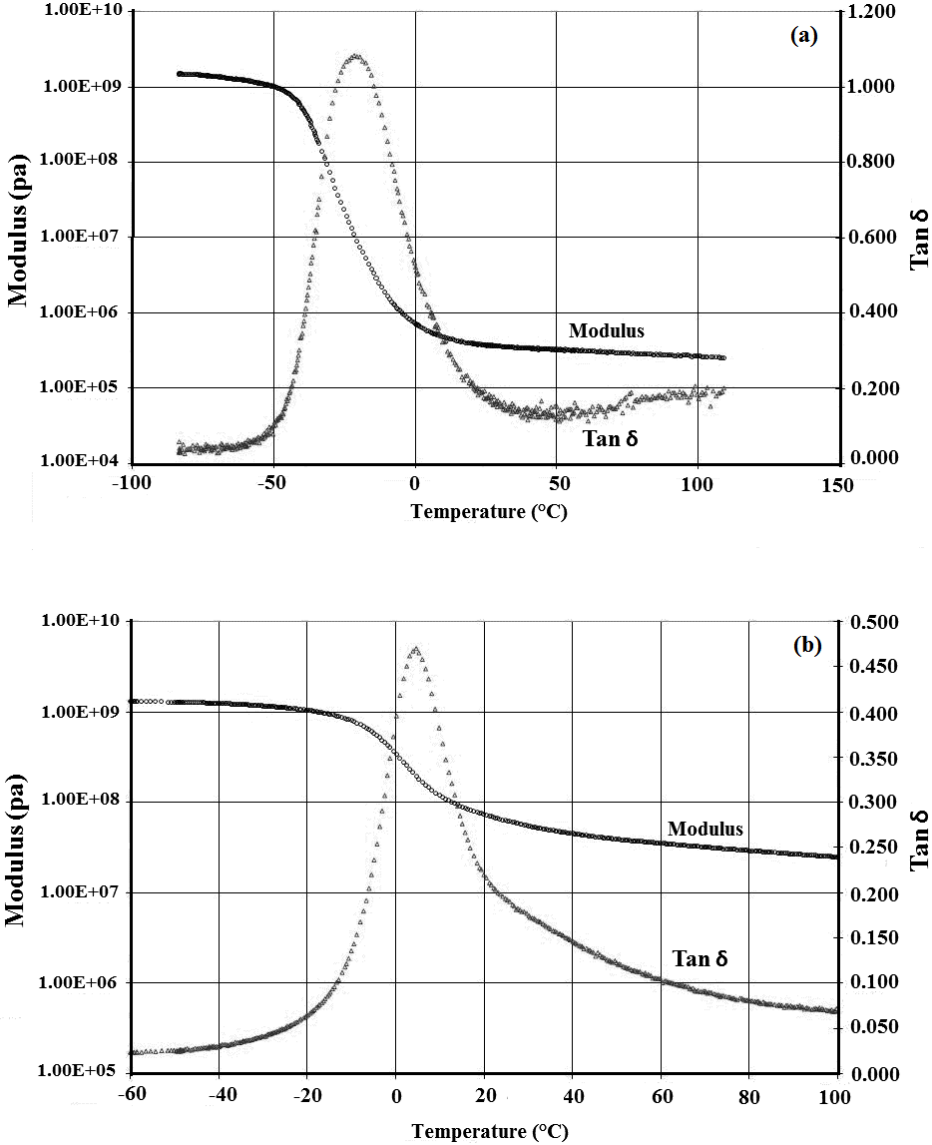
Temperature dependence of storage modulus and loss tangent (tan δ) for (**a**) neat copolymer; and (**b**) synthesized nanocomposite.

DMTA is often used to study relaxations in polymers. Analysis of the storage modulus, the loss modulus and tangent (tan δ) is a key factor in understanding the performance of a sample *versus* temperature [52]. The storage modulus and tan δ of polyacrylate latex and its nanocomposite depended on the temperature (as shown in Figure 6). It was apparent that the storage modulus of the nanocomposite was higher than that of the neat copolymer. In addition, the comparison between the two samples revealed that in ambient temperature and higher, the storage modulus of the nanocomposite was approximately 200-times greater than that of the neat acrylic resin. Furthermore, damping values of the nanocomposite and the neat acrylic resin in the temperature range of 0–50 °C are so close that the flexibility of the neat acrylic resin was equal to that of the synthesized nanocomposite in this range. The glass temperature (T_g_) of the nanocomposite and the neat copolymer were equal to −7 °C and −37 °C, respectively.

### 2.7. Flammability Test

**Figure 7:**
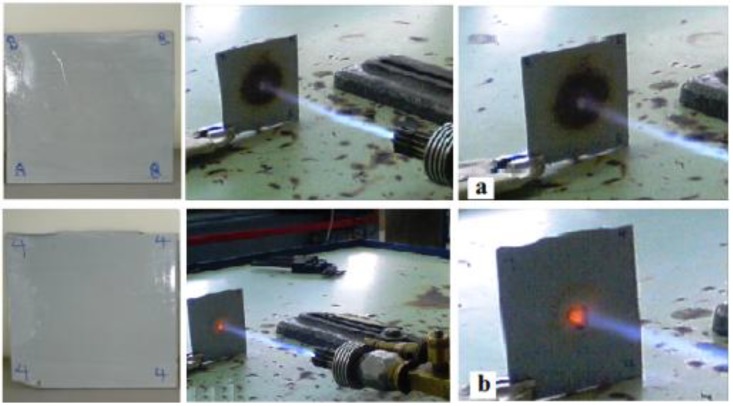
Flammability test for (**a**) neat copolymer; and (**b**) synthesized nanocomposite.

The flammability test was performed to gain a qualitative observation of the effect of the silica encapsulation. The result, as shown in Figure 7, indicates that after being exposed to the flame, the color of the neat copolymer film became brown in a large area around the heated area. However, the color of the polymer nanocomposite showed a good flame resistance, with no or little flame mark around the heated area [53]. In comparison with the neat copolymer, the nanocomposite film was damaged very slightly. Therefore, we can conclude that the acrylic polymer matrix embedded with silica nanoparticles had a superior flame resistance, offering applications in making inflammable paints and coatings.

## 3. Experimental Section

### 3.1. Materials

The silica sol, used in the present study, was Ludox TM-40, purchased from Sigma-Aldrich. The silica sol was a 40 wt% aqueous dispersion of silica nanoparticles with an approximate diameter of 30 nm. Nonyl phenol polyethylene glycol ether (NP-7), as nonionic emulsifier (cloud point < 25 °C), and sodium dodecylbenzenesulfonate (SDBS), as ionic emulsifier, were purchased from Kimiyagaran Emroz Co. (Tehran, Iran) and Kimiyaye Iran Co. (Tehran, Iran), respectively. Methyl methacrylate (MMA) and butyl acrylate (BA) as monomers, ammonium peroxydisulfate as initiator and the other chemicals were purchased from Merck Co. (Darmstadt, Germany).

### 3.2. Preparation of the Nanocomposite

In a typical procedure, 360 g of silica sol was charged into a 1000 mL flask, which was equipped with an oil bath, reflux condenser, a thermometer, a mechanical stirrer with a stainless steel anchor device, a dropping funnel and a nitrogen gas inlet. The reaction vessel was degassed with nitrogen for about 15 min. A solution of ammonium peroxydisulfate (0.5 g) and nonyl phenol polyethylene glycol ether (NP-7) (6 g) in deionized water (90 g) was added dropwise to the silica sol. The dispersion was stirred vigorously with a mechanical stirrer at 60 °C. Afterwards, temperature was raised to 70 °C, and 10 g of the monomer mixture, consisting of MMA (60 g) and BA (140 g), was added to the reactor. The solution was kept at 70 °C for one hour. Then, the remaining part of the monomer mixture and the solution of ammonium peroxydisulfate (0.6 g) and SDBS (16 g) in 140 g of deionized water was fed into the reactor with a constant flow rate slow enough to reach monomer starved conditions. The temperature was fixed at 70 °C during the reaction. In order to complete the polymerization, at the end of the feeding, the content of the reactor was stirred for an additional hour. With this procedure, a high solid content (40%) nanocomposite based on poly(methyl methacrylate-co-butyl acrylate) and silica was synthesized via the emulsion polymerization of the acrylate monomers. This amount of solid content is in the range of the desirable values for industrial applications.

### 3.3. Sample Characterization

Fourier transform infrared spectroscopy spectra were evaluated by a PerkinElmer 2000 FTIR spectrometer operating in the 400–4000 cm^−1^ range.

The particle size distribution of nanocomposite latex was determined by a Malvern dynamic light scattering, Zetasizer HS3000 that enabled us to measure the particle, as well as the molecular, weight.

Transmission electron microscopy studies were performed with the Philips EM208S operated at 100 kV. The morphology and the size of the synthesized nanocomposite latex were assessed using TEM. For TEM analysis, the nanocomposite latex sample was diluted 20-fold with distilled water, stained with OsO_4_. A few drops of the prepared solution were deposited on the carbon-coated copper grids for imaging.

The thermal stability of the nanocomposite and neat acrylic resin was investigated using thermogravimetric analysis (TGA). Before the thermal analysis, in order to separate any possible free dispersed silica inside the composite particles in the latex, the latex samples were coagulated with the concentric sulfuric acid, were washed with the distilled water several times and were filtrated by centrifuge. Subsequently, the samples were dried at 60 °C for two days. The TGA analysis was performed from 30 °C to 600 °C with a heating rate of 5 °C/min using Netzsch STA 409 PC/PG under nitrogen flow.

The dynamic mechanical properties of the nanocomposite and the pure polymer were measured using the dynamic mechanical analyzer (DMA) of Triton/tritec2000 (Triton Technology Ltd, Nottinghamshire, UK) under the tension mode of testing. The samples with 1 mm thickness were tested in an ambient atmosphere in a fixed frequency mode of 1.0 Hz and a heating rate of 5 °C/min. The samples were evaluated in the range of −60 °C to 100 °C.

To determine the flame resistance of the nanocomposite and the neat copolymer, the samples were exposed to oxy acetylene flame (1300 °C) for 3 min. The distance between the samples and the flame was 30 cm.

## 4. Conclusions

Encapsulated silica nanoparticles in acrylic copolymer were synthesized via an *in situ* emulsion polymerization. The method consisted of the surface modification of the nanosilica particles with thermoresponsive nonionic surfactant, which was followed by the emulsion polymerization of the acrylic monomers on the surface of the modified silica. All of the process steps were carried out in a single pot, which simplified the material synthesis. The homogenous dispersion of the nanosilica particles without any agglomeration and their thermal stability were confirmed by TEM, FTIR, TGA and DLS methods. The encapsulation of the silica nanoparticles in the acrylic copolymer elevated the decomposition temperature of the neat copolymer from 341 °C to 373 °C. Furthermore, the DMTA analysis showed the improved mechanical properties of the nanocomposite structure. The produced nanocomposites with the high solid content of silica nanoparticles can be used as a waterborne resin in high thermal resistance paints and coatings.
